# Online tree expansion could help solve the problem of scalability in Bayesian phylogenetics

**DOI:** 10.1093/sysbio/syad045

**Published:** 2023-07-27

**Authors:** Jakub Truszkowski, Allison Perrigo, David Broman, Fredrik Ronquist, Alexandre Antonelli

**Affiliations:** Department of Biological and Environmental Sciences, University of Gothenburg, P. O. Box 461, SE.405 30 Gothenburg, Sweden; Gothenburg Global Biodiversity Centre, Box 461, 405 30 Gothenburg, Sweden; Department of Biological and Environmental Sciences, University of Gothenburg, P. O. Box 461, SE.405 30 Gothenburg, Sweden; Gothenburg Global Biodiversity Centre, Box 461, 405 30 Gothenburg, Sweden; Department of Computer Science and Digital Futures, KTH Royal Institute of Technology, SE.100 44 Stockholm, Sweden; Department of Bioinformatics and Genetics, Swedish Museum of Natural History, P. O. Box 50007, SE.104 05 Stockholm, Sweden; Department of Biological and Environmental Sciences, University of Gothenburg, P. O. Box 461, SE.405 30 Gothenburg, Sweden; Gothenburg Global Biodiversity Centre, Box 461, 405 30 Gothenburg, Sweden; Royal Botanic Gardens, Kew, Richmond, Surrey TW9 3AE, UK; Department of Plant Sciences, University of Oxford, South Parks Road, Oxford OX1 3 RB, UK

**Keywords:** Bayesian inference, MCMC, phylogeny, sequential Monte Carlo

## Abstract

Bayesian phylogenetics is now facing a critical point. Over the last 20 years, Bayesian methods have reshaped phylogenetic inference and gained widespread popularity due to their high accuracy, the ability to quantify the uncertainty of inferences and the possibility of accommodating multiple aspects of evolutionary processes in the models that are used. Unfortunately, Bayesian methods are computationally expensive, and typical applications involve at most a few hundred sequences. This is problematic in the age of rapidly expanding genomic data and increasing scope of evolutionary analyses, forcing researchers to resort to less accurate but faster methods, such as maximum parsimony and maximum likelihood. Does this spell doom for Bayesian methods? Not necessarily. Here, we discuss some recently proposed approaches that could help scale up Bayesian analyses of evolutionary problems considerably. We focus on two particular aspects: *online phylogenetics*, where new data sequences are added to existing analyses, and *alternatives to Markov chain Monte Carlo* (MCMC) for scalable Bayesian inference. We identify 5 specific challenges and discuss how they might be overcome. We believe that online phylogenetic approaches and Sequential Monte Carlo hold great promise and could potentially speed up tree inference by orders of magnitude. We call for collaborative efforts to speed up the development of methods for real-time tree expansion through online phylogenetics.

Bayesian approaches to phylogenetic inference have gained considerable popularity over the past two decades. In Bayesian statistics, prior knowledge and assumptions about the parameters of interest are represented by the prior distribution, which is combined with the likelihood of the data to arrive at the posterior distribution of the parameters. By representing both prior knowledge and posterior estimates as probability distributions, the Bayesian paradigm offers a powerful and flexible modelling approach that allows researchers to quantify the uncertainty of inferences in a principled and intuitive way. The posterior distribution can be used to quantify the uncertainty about a parameter of interest (such as the position of a specific clade) while accounting for its dependency on other parameters (such as the branch lengths across the tree), as opposed to conditioning on specific values of those parameters, as is the case with maximum likelihood. Another key advantage of Bayesian methods over other inference algorithms is the streamlined generation of tree samples containing similarly probable tree hypotheses. These samples can then be analyzed further to study diverse topics, such as the placement of specific clades (Brown and Thomson 2017), species delimitation (Reid and Carstens 2012), or trait diversification (Susoy et al. 2015). These desirable properties have contributed to the growing popularity of Bayesian phylogenetic reconstruction, which has been widely used in diverse fields such as systematics (Cano et al. 2018), infectious disease epidemiology (Nie et al. 2020), palaeontology (Wright et al. 2022), biogeography, and phylogeography (Pedersen et al. 2018). Popular software packages for Bayesian phylogenetic inference, such as MrBayes (Ronquist et al. 2012), BEAST (Suchard et al. 2018), BEAST 2 (Bouckaert et al. 2019), and RevBayes (Höhna et al. 2016) enable biologists to run complex analyses with little to no coding effort.

The main barrier to more widespread adoption of Bayesian phylogenetic methods has been the computational cost of analyzing large data sets. Almost all Bayesian phylogenetic reconstruction methods rely on Markov chain Monte Carlo (MCMC) for sampling from the (approximate) posterior distribution. More specifically, current methods use the Metropolis-Hastings algorithm (Hastings 1970), which constructs a Markov chain that moves through the space of phylogenetic trees in such a way that it is guaranteed to eventually converge to the posterior distribution. Unfortunately, such Markov chains are often slow to converge and it is generally not possible in advance to estimate the amount of time necessary to generate a sufficient number of samples from the posterior distribution. In practice, MCMC works efficiently on data sets comprising dozens up to several hundreds of sequences, but remains prohibitively slow for most large data sets (Zhang et al. 2020).

A characteristic aspect of modern phylogenetic inference is the frequent combination of old and new data in the same analysis. In molecular systematics, researchers typically want to compare a small number of newly sequenced species to a large number of well-studied and previously sequenced species to understand the relationship of the new species to established taxonomic groups (Giaretta et al. 2019; Lin et al. 2022; Rush and Aime 2013). In molecular epidemiology, researchers aiming to understand the evolution of SARS-CoV-2 compare newly sequenced viral genomes to the database of already sequenced genomes, which likely have been analyzed under similar assumptions. This creates an opportunity for re-using the results of previous analyses to quickly obtain updated inferences without having to relaunch the analysis from scratch, which often takes days or weeks to complete. In the past few years, this analytical paradigm has come to be known as *online phylogenetics* (Fourment et al. 2018; Gill et al. 2020; Bouckaert et al. 2022), as opposed to *offline* analyses, in which all the computation is performed from scratch without taking advantage of previous analyses.

Online phylogenetics has recently attracted renewed interest due to its potential to radically speed up phylogenetic inference on today’s large and continually expanding sequence data sets. This has led some researchers to propose methods that adapt existing MCMC approaches to online analyses (Gill et al. 2020; Bouckaert et al. 2022). Others have developed online algorithms based on Sequential Monte Carlo (SMC) (Fourment et al. 2018), an alternative framework for Bayesian data analysis that is well-suited for online inference.

Here, we first briefly survey existing approaches to online Bayesian phylogenetic inference, including alternatives to the MCMC method for estimating the posterior distribution. We then discuss the challenges that must be addressed before these methods can reach widespread popularity. Finally, we suggest possible directions for future research. For a recent discussion of scalability in offline analyses, see Fisher et al. (2022).

## Online phylogenetic methods

**Figure 1:**
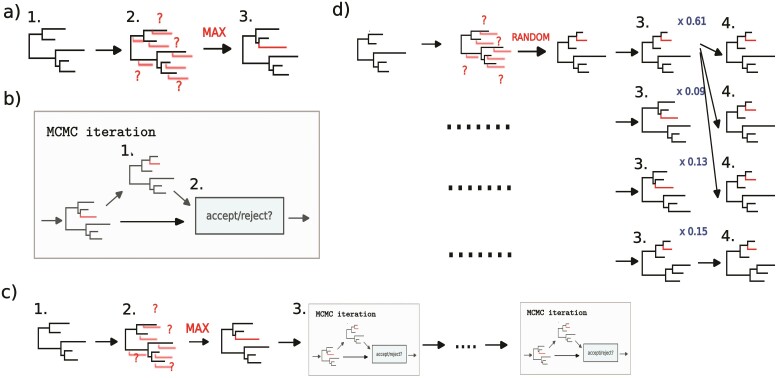
Current methods for online phylogenetic analyses. All methods start from a reasonable tree estimate, which then needs to be improved based on the information contained in the new sequence. a) Maximum likelihood phylogenetic placement. Maximum likelihood methods use the existing phylogeny 1) to compute the likelihood of attaching a new sequence onto every possible branch 2) and choose the attachment that maximises the likelihood of the resulting tree 3). This method does not update the tree beyond the added branch. If repeated many times, this can significantly reduce the accuracy of the tree. b) MCMC iteration. An MCMC iteration consists of randomly proposing a small change to the topology or branch lengths of the current tree 1) which is then probabilistically accepted or rejected based on the posterior ratio between the proposed and the current tree 2). c) Online MCMC (Gill et al. 2020; Bouckaert et al. 2022). The starting point of an online MCMC analysis is informed by a previous MCMC run (1). As in the case with maximum likelihood, this method adds one or more sequences to a tree sampled from a previous MCMC run (2). After the sequences are added, the resulting tree serves as the starting point for another MCMC run; MCMC iterations are applied to the tree until a pre-defined convergence criterion is met(3, which is the same as an MCMC iteration as described in (b) but carried out successively). d) Online phylogenetic SMC (Fourment et al. 2018). SMC also samples a population of trees from the previous run 1), it then attaches a new sequence randomly to each of these trees based on the approximate posterior probability of possible attachments 2). Each tree is then assigned a weight proportional to the ratio of the likelihood of the tree with the sequence inserted against the likelihood of tree without the sequence 3)—see Equation (1). The trees are then resampled proportionally to their weights 4).

In this section, we give an overview of methods that update existing analyses with new sequence data. Figure 1 illustrates the methods discussed here.

### Phylogenetic Placement

The oldest group of online phylogenetic methods involves inserting new sequences into an existing reference tree. These methods take as input a single gene tree inferred from an existing multiple sequence alignment and a set of novel query sequences aligned to that alignment. The task is then to find the optimal placement of each sequence on the tree.

While the idea of building trees by sequentially adding taxa has a long history (Farris 1970; Schmidt et al. 2002; Truszkowski et al. 2012), it was only in the early 2010s that researchers developed methods specifically designed for phylogenetic placement. Pplacer (Matsen et al. 2010) first evaluates the likelihood of attaching a new sequence in the middle of every branch of the reference tree. It then optimizes the attachment point on the branch and the length of the pendant branch to produce the most likely attachment or an approximate posterior distribution of attachments. EPA and EPA-ng (Berger et al. 2011; Barbera et al. 2019) follow a similar strategy, though they also re-optimize the lengths of the two branches that arise from subdividing a branch of the reference tree onto which the new sequence is inserted. LSHPlace (Brown and Truszkowski 2013) uses locality-sensitive hashing (Indyk and Motwani 1998) to efficiently find branches that are likely to yield high-likelihood placements, without the need to evaluate every branch in the reference tree. Even faster hashing-based methods have recently been developed for the placement of large numbers of environmental sequences without the need for alignment (Linard et al. 2019). Other recent efforts to increase the efficiency of phylogenetic placement involve distance methods (Balaban et al. 2020) and divide-and-conquer strategies (Balaban et al. 2021; Koning et al. 2021).

Recently, phylogenetic placement has received renewed attention in the context of large-scale phylogenetic analyses during the COVID-19 pandemic. With up to 1000s of genomes being sequenced every day (Brito et al. 2021), fast methods are needed to take advantage of all the new data. UShER (Turakhia et al. 2020) uses a succinct data structure to keep track of the differences between entire SARS-CoV-2 genomes. This data structure is then used to quickly infer the placement of a new genome by minimizing the parsimony score of the resulting tree.

Crucially, phylogenetic placement methods do not seek to improve our estimate of the relationships between previously included sequences, except possibly re-estimating the length of the branches adjacent to the inserted sequence, as is the case in the Evolutionary Placement Algorithm (EPA). As a result, the accuracy of trees inferred in this way will generally be lower than trees inferred by running a new analysis from scratch, and the difference between the two will tend to increase with each added sequence. One possible exception might be extremely densely sampled data sets with very low evolutionary distances between sequences, as is the case with SARS-CoV-2 genomes.

### Online Phylogenetic MCMC

Markov chain Monte Carlo (MCMC) is by far the most popular paradigm for Bayesian phylogenetic inference. MCMC can be applied to a wide range of models and is theoretically guaranteed to produce samples from the posterior distribution if the chain is run for a sufficient number of iterations—see (Yang 2014) for an extensive review. The continued dominance of MCMC is also due to the existence of well-maintained implementations, such as BEAST 2 (Bouckaert et al. 2019), MrBayes (Ronquist et al. 2012), RevBayes (Höhna et al. 2016), and PhyloBayes (Lartillot et al. 2013). Adapting these implementations for online inference would make online phylogenetics accessible to a large community of researchers already familiar with these tools.

Gill et al. (2020) introduced a heuristic online MCMC method intended for phylodynamic inference. The method samples a tree from a previous MCMC run and inserts each new sequence into that tree by attaching it as a sister sequence to the closest sequence already in the tree. The resulting tree is then used as a starting tree for a new MCMC run. The authors show that such an initialization strategy yields greatly improved convergence times. However, it is not clear how such a deterministic initialization strategy impacts commonly used methods for MCMC convergence diagnostics, which generally assume that MCMC chains are initialized by drawing from a distribution that is more dispersed than the target posterior (Gelman and Rubin 1992).

More recently, Bouckaert et al. (2022) proposed a more parallelizable online MCMC method. The method first samples a number of trees from a previous MCMC run and inserts new sequences into them in a way that approximately maximizes the posterior probability of the resulting trees. Each tree is then subjected to a number of local MCMC steps, where MCMC proposals are only performed in the neighborhoods of the newly inserted sequences, with the rest of the tree kept intact. Finally, the method performs a number of standard MCMC iterations affecting the whole tree. These steps are performed independently for each tree sampled from the previous run, so they can be easily parallelized. The authors demonstrate the performance of the method on a SARS-CoV-2 data set where 730 full genomes are analyzed based on an initial analysis of 320 full genomes. As in the case of Gill et al. (2020), it is not entirely clear how to monitor the convergence of this approach, which makes rigorous performance comparison challenging. The authors caution against overly trusting any particular convergence diagnostic.

### Online Phylogenetic Sequential Monte Carlo

The primary disadvantage of MCMC methods is their slow convergence times, particularly for large data sets. For data sets comprising large numbers of taxa, MCMC methods struggle with exploring the enormous space of possible trees. Even for moderate numbers of taxa, the combination of large amounts of sequence data and conflicting phylogenetic signals may give rise to challenging posterior distributions with multiple high-probability areas separated by deep “valleys” of low probability, which causes MCMC algorithms to get trapped in the same area for prolonged periods of time (Mossel and Vigoda 2005). The slow convergence of MCMC is a pervasive problem in many fields where Bayesian data analysis is used (Papamarkou et al. 2022). As a result, researchers have shown a renewed interest in alternatives to MCMC in recent years.

Sequential Monte Carlo (SMC) is perhaps the most well-known alternative to MCMC. In its most common form, an SMC algorithm samples from a sequence of posterior distributions given increasing amounts of data (Fig. 1d). At every iteration, it maintains a population $\left\{x_{n}^{i}|i=1\ldots N\right\}$ of trees, also called *particles*, which approximates the posterior distribution conditional on the data $y_{1},\ldots,y_{n}$ processed so far. Given a new data point $y_{n+1}$, the algorithm updates the samples using a *proposal distribution* $q(.|x_{n}^{i},y_{n+1})$ to adapt the posterior to new data. The proposal distribution is usually chosen to approximate the new posterior distribution, while being computationally easy to sample from. The particles are then re-weighted to account for the difference between the proposal and the new posterior


$$w_{n+1}^{i}=\frac{p(y_{n+1},\ldots ,y_{1}|x_{n+1}^{i})p(x_{n+1}^{i})}{q(x_{n+1}^{i}|x_{n}^{i},y_{n+1})p(y_{n},\ldots ,y_{1}|x_{n}^{i})p(x_{n}^{i})}$$ (1)

Finally, a *resampling* step is performed where the particles are resampled with probability proportional to their weights. This is done to remove low-weight particles and duplicate high-weight particles to make more efficient use of the computational resources.

SMC algorithms are naturally suited to process data in an online fashion, which is invaluable in many modern applications where data sets are continually being updated. In phylogenetics, this paradigm has been introduced as Online Phylogenetic Sequential Monte Carlo (OPSMC) by Fourment et al. (2018). OPSMC maintains an approximate posterior sample of trees on a set of $n$ taxa. Upon arrival of a new sequence, the proposal distribution first samples a likely edge onto which the new sequence should be inserted for each tree in the sample. It then samples the attachment point on the chosen edge and the length of the newly created pendant branch. The particles are then re-weighted and resampled as described above. Through experiments on simulated data, Fourment et al. (2018) show that choosing the right proposal distribution is crucial to the computational efficiency and accuracy of OPSMC. In their experiments, the most efficient proposals are those based on likelihood and parsimony scores of the proposed trees. The authors show that the most efficient proposals yield algorithms that are orders of magnitude faster than corresponding MrBayes analyses, while having similar accuracy. One limitation of OPSMC is the accumulation of resampling error in subsequent steps, which limits the number of sequences that can be added to an existing analysis without further refinement of the procedure.

## Challenges

Both online MCMC and online SMC methods of phylogenetic inference hold great untapped potential. However, we see 5 specific challenges that need to be overcome for them to reach their full potential in terms of scalability and ease of use.

### Challenge 1: Tackling Path Degeneracy in SMC

SMC usually requires multiple resampling steps to reach the target distribution. Each one of these increases the variance of clade posterior probability estimates produced by the algorithm and reduces the diversity of samples as some trees do not get resampled whereas others are resampled many times. When many sequences are added, this results in the accumulation of sampling error over time and the emergence of “founder effects,” where the subtrees induced on the earliest added taxa are nearly identical across the particles. This phenomenon is known as *path degeneracy* and is mathematically analogous to founder effects in population genetics (Chopin et al. 2020). Fourment et al. (2018) indicate that this limits the number of SMC iterations to around $5$.

Path degeneracy is a well-known problem in the SMC literature and several remedies have been proposed. *MCMC rejuvenation* (Gilks and Berzuini 2001) involves performing a limited number of MCMC moves after each SMC iteration. The MCMC moves counteract path degeneracy at the cost of increased computation time. *Rejection control* (Liu 2001) performs additional SMC proposals in order to limit the probability of eliminating a particle. To our knowledge, none of those ideas have been explored in the phylogenetics literature; we think these techniques could potentially extend the number of sequences that can be added well beyond the current limits.

### Challenge 2: Balancing Local Versus Global Tree Modifications

Both phylogenetic placement and OPSMC can be seen as *local* methods—once a new sequence is placed into the tree, these methods update only the local parameters, such as branch lengths in the vicinity of the new sequence. While it is clear that this leads to computational savings, the tradeoff between accuracy and scalability is not very well understood. Bouckaert et al. (2022) argue that global updates are necessary for accurate inference based on two observations. First, most modern phylogenetic analyses use substitution models with global parameters, such as transition/transversion ratio or the shape parameter for rate distribution. The authors argue that adding even one sequence changes our belief about likely substitution model parameters, which might have an impact across the tree. Second, the authors argue that classical distance methods, such as UPGMA (Sokal 1958) or Neighbor Joining (Saitou and Nei 1987; Studier and Keppler 1988), can potentially produce very different results after adding an extra sequence to the data set, which they view as evidence that phylogenetic reconstruction is an inherently global problem.

We think that both of those claims require further investigation. While it is clear that the choice of substitution model can globally influence the tree posterior, the examples provided by Bouckaert et al. (2022) involve either very small numbers of taxa or large numbers of added sequences. It is thus possible that local methods could still be effective when the number of added sequences is not too high relative to the number of sequences already in the tree as the impact of the new sequences on substitution model parameters might then be negligible.

While we are not aware of studies investigating the impact of adding new sequences on distance methods, the relationship between distance estimates and inferred trees has been extensively studied. Classical results (Atteson 1999; Mihaescu et al. 2009) show that NJ produces correct trees if all errors are less than half the length of the shortest branch in the true tree. Moreover, NJ is known to require very long sequences to be accurate on data sets with many distant sequence pairs (Lacey and Chang 2006), so it might not be representative of more efficient methods. Indeed, some theoretical results (Erdös et al. 1999; Brown and Truszkowski 2012) suggest that local changes to tree structure can be sufficient for accurate reconstruction if enough data are available.

The possibility that sequence additions have global impacts on phylogenetic inference has serious implications for phylogenetics beyond online analyses—for most taxonomic groups, our sampling of taxa is likely to be incomplete as it is clear that a large number of species remain scientifically undescribed (Mora et al. 2011). Quantifying the impact of taxon sampling on the inferred relationships between major clades would improve our understanding of reliability of phylogenetic inferences across the tree of life.

### Challenge 3: Managing Memory Usage and Parallelization

Phylogenetic inference for large data sets requires a considerable amount of main memory. Most of the memory complexity is due to the need to store the partial results of likelihood computations for all internal nodes in the tree and for all columns in the alignment. Commonly used offline methodologies, such as maximum likelihood or MCMC, only require one or several trees to be stored at any one time in the execution of the algorithm. In contrast, both OPSMC and the online MCMC of Bouckaert et al. (2022) maintain a population of hundreds to thousands of trees. When implemented naively, an online algorithm would thus require orders of magnitude more memory than a standard offline MCMC analysis. This is unacceptable for large data sets.

An alternative approach is to recompute the conditional probabilities as needed by the algorithm and discard them when moving to another batch of trees. While this removes the need for large amounts of main memory, it increases the running time of the method, which creates a tradeoff between the amount of memory required and the running time of the analysis. This tradeoff becomes even more pronounced for parallel architectures, as the amount of memory impacts the number of computations that can be executed in parallel. Optimizing the computational performance of online phylogenetic methods will require exploring how to best use the limited memory available and how to schedule the computations to leverage the processing capacity of modern parallel architectures such as graphics processing units.

### Challenge 4: Combining MCMC and SMC

SMC and MCMC algorithms developed independently up until the mid-2000s. In 2006, Del Moral et al. (2006) proposed *SMC samplers*, which enable researchers to easily reuse MCMC proposal distributions within an SMC algorithm. This is particularly important in phylogenetics as it enables researchers to leverage vast existing literature on MCMC proposal distributions while taking advantage of the benefits of SMC, such as unbiased estimates of marginal likelihood and more robust convergence diagnostics. This possibility was investigated by Wang et al. (2020), who reported improved marginal likelihood estimates compared to MCMC-based methods. Combining MCMC and SMC methods is an active topic of research within Bayesian statistics; researchers are particularly interested in increasing computational efficiency (Dau and Chopin 2020) and inference in high dimensions (Chopin et al. 2020). An interesting research question is whether the approach of Bouckaert et al. (2022) could be reformulated as an SMC sampler.

Particle MCMC (Andrieu et al. 2010) is another class of methods that alternate between SMC and MCMC steps. In phylogenetics, they have been used to jointly estimate global model parameters and tree topologies (Endo et al. 2019; Wang and Wang 2021), which has been difficult to achieve using pure SMC methods. It remains to be seen whether these methods could be useful for online analyses.

### Challenge 5: Software Implementation

Both MCMC and SMC algorithms require considerable implementation and testing effort, despite the existence of efficient open-source implementations of phylogenetic likelihood calculations, such as BEAGLE (Ayres et al. 2019) and PLL (Flouri et al. (2015)). In recent years, the Bayesian statistics community has shown increasing interest in automating some of the steps involved in the development of Bayesian inference algorithms through the use of *probabilistic programming languages* (PPLs), where a probabilistic model is expressed as a program written in a dedicated language (Goodman and Stuhlmüller 2014; Carpenter et al. 2017). The PPL system then synthesizes the code of an SMC or MCMC algorithm based on the model description. These approaches have the potential to reduce development time and greatly increase the reproducibility of Bayesian analyses.

An early example of the usefulness of PPLs in phylogenetics was provided by Ronquist et al. (2021) who used them to estimate diversification model parameters. Recent attempts to improve the efficiency and flexibility of PPLs have used problems in phylogenetics as motivating examples (Bouchard-Côté et al. (2022); Lundén et al. (2022)). We think that PPLs are a promising paradigm for Bayesian phylogenetics, though more effort is required to adapt existing frameworks to complex data types such as tree topologies.

## Conclusions

Online Bayesian analyses are likely to become increasingly popular as sequence data rapidly grows in size and taxonomic coverage. We consider the SMC and MCMC approaches discussed in this paper to have complementary strengths. SMC methods promise reduced running times and unbiased marginal likelihood estimates. On the other hand, current online SMC methods are limited by the small number of sequences that can be added and might not be able to efficiently update global parameters. MCMC methods are better suited to global updates, but their running times are substantially higher and it is not yet entirely clear how to monitor their convergence in an online setting. To the best of our knowledge, a thorough comparison of existing methods is still missing from the literature. We argue that as techniques from both paradigms are further developed, their synergies need to be explored rather than kept apart. A fruitful path forward for online phylogenetic methods lies in finding creative ways to combine and augment existing methods and rigorously evaluate them. The resulting improvements in scalability will expand the scope of applications and contribute to the wider adoption of Bayesian phylogenetic methods across diverse research communities.
